# A machine learning-based method for feature reduction of methylation data for the classification of cancer tissue origin

**DOI:** 10.1007/s10147-024-02617-w

**Published:** 2024-09-18

**Authors:** Marco A. De Velasco, Kazuko Sakai, Seiichiro Mitani, Yurie Kura, Shuji Minamoto, Takahiro Haeno, Hidetoshi Hayashi, Kazuto Nishio

**Affiliations:** 1 Department of Genome Biology, Faculty of Medicine, Kindai University, Ohnohigashi 377-2, Osaka-Sayama, 589-9511 Japan; 2 Department of Medical Oncology, Faculty of Medicine, Kindai University, Osaka-Sayama, Japan; 3 Department of Molecular Tumor Pathobiology, Kindai University Graduate School of Medical Sciences, Osaka-Sayama, Japan

**Keywords:** Cancer of unknown primary, Deep learning, Methylation profiles, Primary organ classification, Gradient boosting

## Abstract

**Background:**

Genome DNA methylation profiling is a promising yet costly method for cancer classification, involving substantial data. We developed an ensemble learning model to identify cancer types using methylation profiles from a limited number of CpG sites.

**Methods:**

Analyzing methylation data from 890 samples across 10 cancer types from the TCGA database, we utilized ANOVA and Gain Ratio to select the most significant CpG sites, then employed Gradient Boosting to reduce these to just 100 sites.

**Results:**

This approach maintained high accuracy across multiple machine learning models, with classification accuracy rates between 87.7% and 93.5% for methods including Extreme Gradient Boosting, CatBoost, and Random Forest. This method effectively minimizes the number of features needed without losing performance, helping to classify primary organs and uncover subgroups within specific cancers like breast and lung.

**Conclusions:**

Using a gradient boosting feature selector shows potential for streamlining methylation-based cancer classification.

**Supplementary Information:**

The online version contains supplementary material available at 10.1007/s10147-024-02617-w.

## Introduction

Cancer of unknown primary (CUP) is a poor prognostic malignancy with an unknown primary site and histologically known metastases[1–4]. Most patients with CUP, except for about 20% with favorable prognostic factors, receive empiric chemotherapy including platinum-taxane regimens [5, 6], and experience a median overall survival (OS) of about 6–12 months and short survival times [7–9]. Optimizing drug therapy based on primary organ estimation could potentially improve outcomes for patients with poor prognosis CUP [3]. Molecular profiling of CUP using tools such as gene expression, DNA methylation, and somatic mutation profiling has been used to predict the tissue origin of CUP. However, contrary to expectations, using such methods to guide site-specific therapy was found not to improve OS compared with empiric chemotherapy [10–13]. It is possible that assigning CUP tumors to site-specific therapies based on molecularly predicted profiles may not be sufficient to improve treatment outcomes. However, it is also possible that previous classifiers, particularly those based on transcriptomic profiles, may have failed to accurately ascertain the proper tissue of origin.

Methylation is a regulatory mechanism of gene expression in which a methyl group (CH_3_) is bound to a base of DNA. Methylation suppresses gene expression and is involved in many biological processes, including cell differentiation and cancer development. Methylation usually occurs in the CpG islands, a region of DNA in which cytosine (C) and guanine (G) are adjacent to each other. The literature suggests that DNA methylation patterns exhibit organ-specific patterns [14]. Recently, Liu et al. constructed a machine-learning algorithm based on methylation profiles to identify tissue origin with promising results [15]. Hoadley et al*.* proposed a method to track the origin of 12 cancer types based on methylation and copy number variation [16]. To the best of our knowledge, no systematic comparison has yet been made between them. However, it is estimated that 80% of the human genome is methylated—thus, the amount of data generated from methylation profiling is quite large and can contain millions of methylation sites [17]. Popular platforms such as the Illumina Human Methylation 450 k cover over 450,000 methylation sites, within and outside CpG islands. Targeted sequencing platforms are also a rapid and cost-effective means of identifying known genetic alterations in selected gene sets and have been widely adopted in cancer clinical practice. However, this approach requires selecting the most appropriate features suitable for a prediction model.

In this study, we utilize site-specific methylation to develop a classifier that estimates primary tumor site based on methylation patterns of tumor tissues. The classifier is constructed using a focused set of 100 CpG sites selected through machine learning in a subset of cancers. We compared the utility of using embedded machine learning methods to extract informative CpG sites that could be used to train various types of machine learning models with statistical filtering methods. Lastly, we perform an unsupervised analysis of the CpG sites selected by Gradient Boosting.

## Materials and methods

### Methylation data set

Methylation data from Illumina Infinium Methylation 450 k array from a subset of TCGA cases were used and were obtained through the NCI Genomic Data Commons (GDC) portal (https://portal.gdc.cancer.gov/). The TCGA data set comprised DNA methylation β values of 488,575 CpG sites (features) from 890 samples, including the following 10 cancer types: breast invasive carcinoma (BRCA) 179 patients, colon adenocarcinoma (COAD) 111 patients, glioblastoma multiforme (GBM) 17 patients, head and neck squamous cell carcinoma (HNSC) 9 patients, kidney renal papillary cell carcinoma (KIRP) 167 patients, lung adenocarcinoma (LUAD) 163 patients, lung squamous cell carcinoma (LUSC) 119 patients, rectum adenocarcinoma (READ) 71 patients. sarcoma (SARC) 33 patients, and stomach adenocarcinoma (STAD) 21 patients. Each cancer type had its patient data randomly divided into training and test data sets using a 70/30 split.

### Data preprocessing, feature selection, and prediction modeling testing

**Fig. 1:**
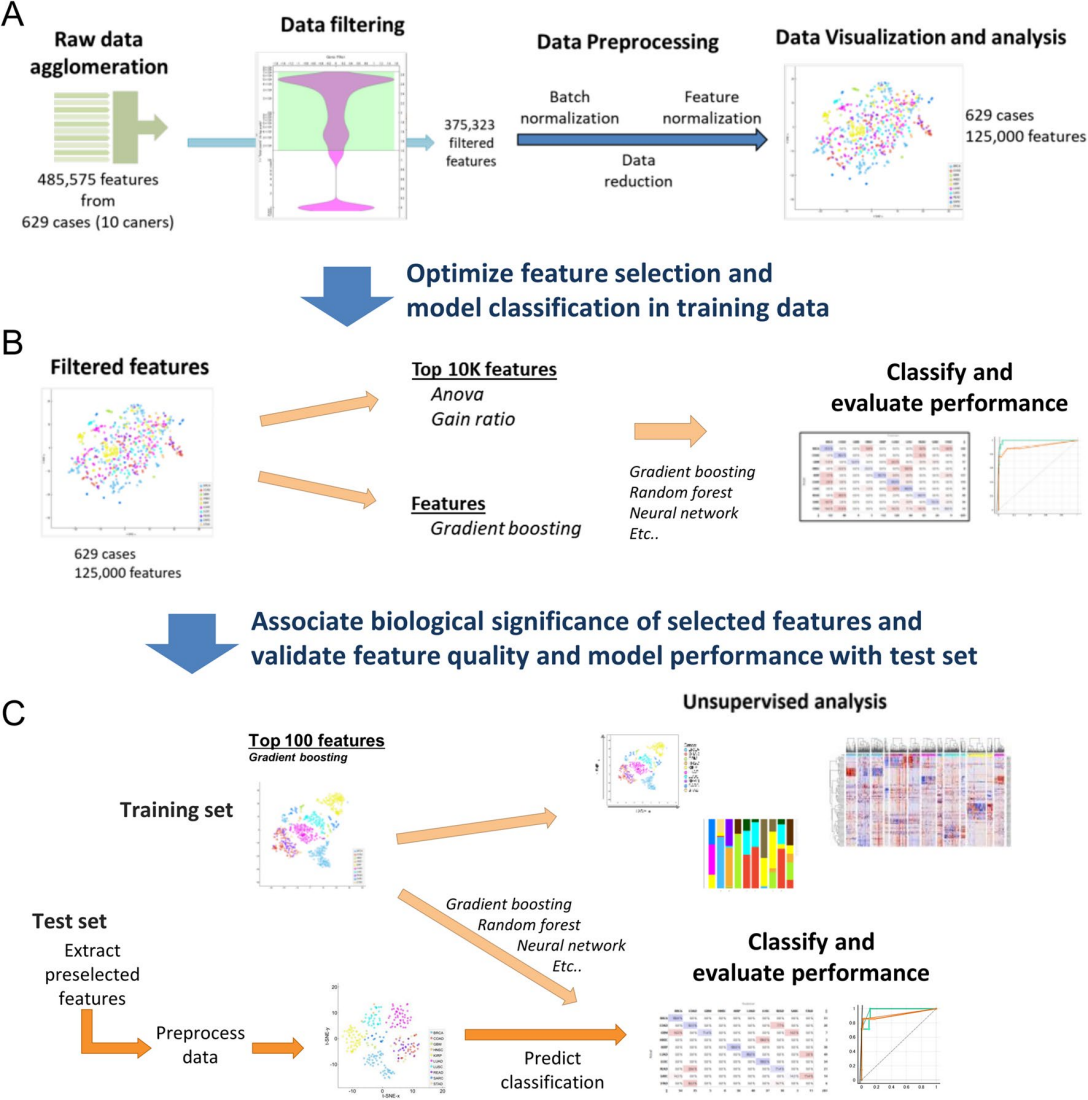
Flowchart of the study process. Methylation data from The Cancer Genome Atlas (TCGA) were used to build a prediction model for determining cancer type. The process consisted of data preprocessing (**A**), feature selection (**B**), and validation (**C**)

Data were processed and analyzed in Orange v3.32, a Python-based machine learning and data mining suite [18]. For the training set, raw data from 629 cases were collected as part of the overall research flow and preprocessing (Fig. 1A). Data preprocessing consisted of compiling data sets, removing infrequent, or zero measurement data followed by batch normalization. Data were then trimmed by selecting 125,000 most variable features based on the mean standard deviation and visualized using t-distributed Stochastic Neighbor Embedding (t-SNE). The remaining 125,000 features were ranked, and the top 10,000 features were selected based on the analysis of variance (ANOVA) or Gain Ratio 17 scores [19], or the top 100 features ranked by gradient boosting as feature scores (Fig. 1B). The classification models used were included in the following packages, Scikit-learn, XGBoost, CatBoost, and LIBSVM within Orange. Models were tested using stratified five-fold cross-validation sampling. For each training test run, various performance metrics were calculated on the test data set, including model accuracy, goodness of fit, repeatability, and F1 score. Predicted confidence scores were also calculated and compared to evaluate the performance characteristics of each model for the actual nuclear organs.

### Test set validation

Features selected from the gradient boosting scoring classifier were extracted from the test set case and preprocessed to correct for batch effects (Fig. 1C). Predicted confidence scores were calculated from Gradient Boosting and compared with other prediction models.

### Unsupervised analysis

Orange v3.32 software was used to perform unsupervised analyses on the 100 features selected from Gradient Boosting scoring. We used the Louvain method for community detection to identify and extract non-overlapping communities within the data. The data was then visualized in 2D plots using t-SNE and a Manhattan distance metric. Correlation of CpG sites was visualized using a clustered distance map generated using Pearson correlation coefficients. For the heatmaps, data were clustered using Euclidean distances and the Ward linkage method, and columns were split according to the Louvain cluster or cancer type.

## Results

### Exploratory data analysis and feature selection

Our primary was to determine if we could develop a model to predict tumor origin based on the methylation status from a reduced number of CpG sites. Given that several factors could impact the quality and utility of the selected features, we aimed to test our approach using a subset of samples from selected cancer sites. The cancer sites were selected based on a range of cancer prevalence, cancer heterogeneity, anatomical location, and biological similarities. We also aimed to establish the minimum case size required to extract informative features for classification. Thus we used unbalanced-sized data sets from 10 cancer types from The Cancer Genome Atlas (TCGA). Methylation data from the Illumina Infinium Human Methylation 450 k platform were used and the TCGA data set contained DNA methylation β values for 485,575 CpG sites from 890 samples, which included 10 cancer types. Patient data from each cancer type was randomly divided into training and test data sets using a 70/30 split (629 and 261 samples, respectively) to ensure an adequate representation of cancer types from unbalanced data sets. An overview of the analysis pipeline is shown in Fig. 1. Our goal was to extract a minimal feature set, which means that we were looking for features that would be the most informative. Based on the large amount of data available we first trimmed the data by eliminating approximately the bottom 25% of features that contained underrepresented or missing values (Fig. 1A). We next proceeded to perform data preprocessing, which included the removal of batch effects to reduce bias from nonbiological factors or other related artifacts (Supplementary Fig. S1). Large data sets can be difficult to work with and pose challenges when working with prediction models, which include data storage, computational power, and statistical challenges, including scalability, high dimensionality, noise, and spurious correlations [20, 21]. Thus, trimmed the data by two-thirds and only kept the most variable features based on mean variance. We then used the remaining pool of 125,000 CpG sites to extract the most informative features that could be used for prediction modeling. For this, we compared three different approaches of feature selection, two filter methods (analysis of variance [ANOVA]) [22] and Gain Ratio [19], and Gradient Boosting [23] as an ensemble machine learning algorithm. ANOVA is a statistically-based filter method that ranks features based on significant group differences. Information Gain is another feature ranking approach that ranks subsets of features based on high information gain entropy [24]. Gain ratio is a variation of Information Gain and was developed to reduce the bias of Information Gain on highly branched predictors [19]. Gradient Boosting is a widely used technique in machine learning. Gradient Boosting is a decision tree ensemble algorithm that is particularly suited for the regression and classification of tabular data [23].

### The prediction accuracy of features selected using statistical and filter methods

**Fig. 2:**
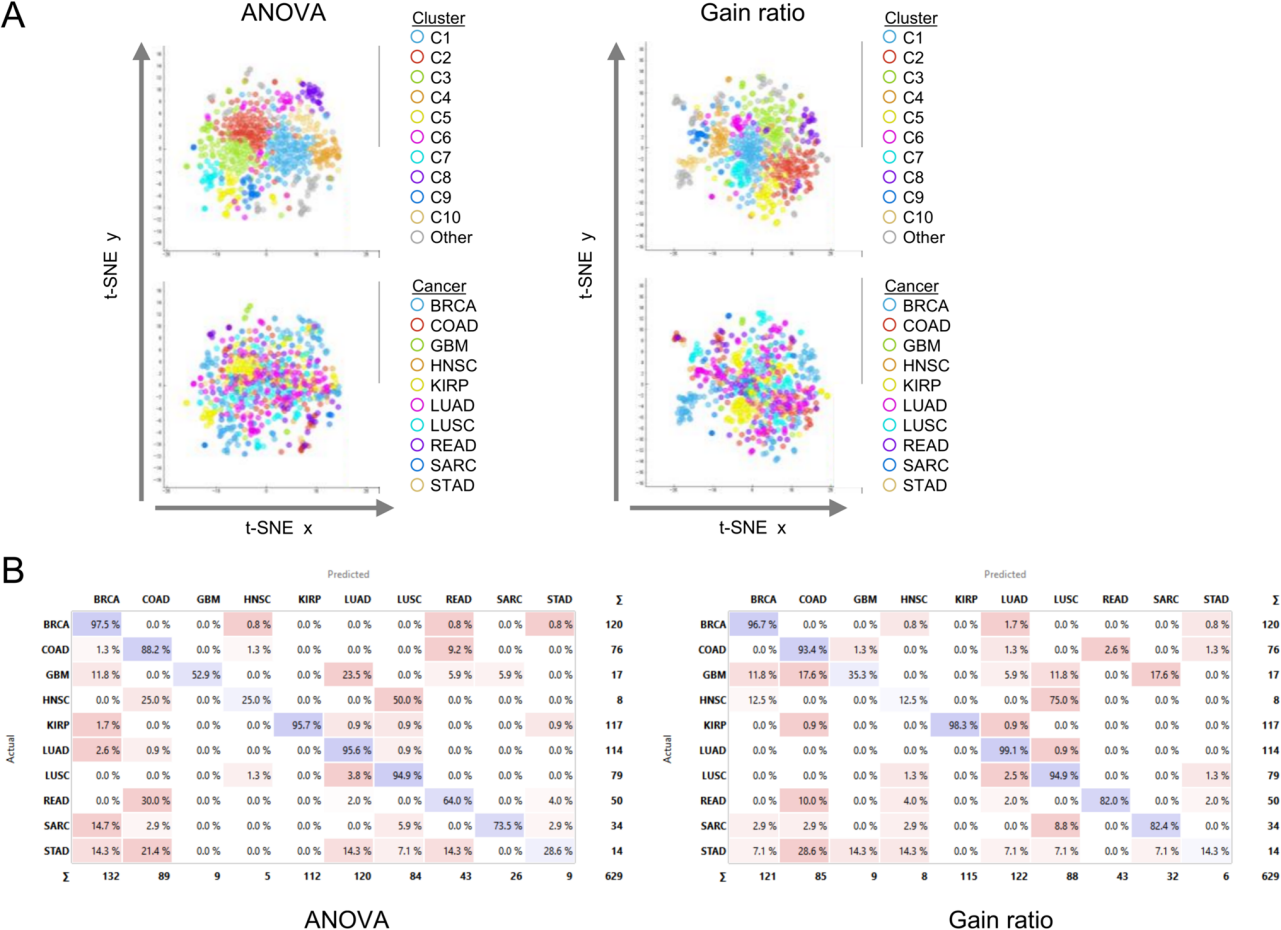
Evaluation of the prediction model using features selected by ANOVA or Gain Ratio. **A** Visualization of data using t-distributed stochastic neighbor embedding (t-SNE) of patients (n = 629) based on the methylation of CpG sites selected by ANOVA or Gain ratio. The Louvain method of community detection was used to identify patient clusters. Colors were assigned according to cluster (top panels) or cancer type (bottom panels). **B** Confusion matrices showing the percentage of patients actually predicted by Gain Ratio classifier trained features selected by ANOVA or Gain Ratio. *BRCA* Breast invasive carcinoma, *COAD* Colon adenocarcinoma, *GBM* Glioblastoma, *HNSC* Head and neck squamous cell carcinoma, *KIRP* Kidney renal papillary cell carcinoma, *LUAD* Lung adenocarcinoma, *LUSC* Lung squamous cell carcinoma, *READ* Rectum adenocarcinoma, *SARC* Soft tissue sarcoma, *STAD* Stomach adenocarcinoma

For ANOVA and Gain Ratio, we extracted the top 10,000 features which represent approximately 2% of the original data or 8% of the trimmed data (Supplementary Tables S1 and S2). We also wanted to determine the similarity of the selected features and determine whether distinct groups or clusters existed within the dataset. For this, we used Louvain clustering as an unsupervised, agglomerative method to identify clusters [25]. ANOVA selection yielded 16 clusters, while Gain Ratio selection resulted in 17 clusters. Two-dimensional (2D) t-distributed Stochastic Neighbor Embedding (t-SNE) was used to visualize patients and associate Louvain clusters with cancer types. While features selected by ANOVA showed better-defined Louvain clusters, features selected by Gain Ratio appeared to show better overlap between Louvain clusters and cancer types (Fig. 2A).

Next, we determined the classification and predictability potential of features selected by ANOVA and Gain Ratio across the 10 cancer types. For this, we used fivefold cross-validation to evaluate the classification performance with several popular machine learning algorithms (refer to Table 1). When considering the goodness of fit (positive predictive value or precision) as the evaluation metric, the top three models for features selected by ANOVA were Gradient Boosting, Random Forest, and AdaBoost, with respective goodness of fit values of 0.876, 0.703, and 0.599 across all classes. The performance of features selected by the Gain Ratio yielded similar results with the ranking of the models but showed a slight improvement with the evaluation metrics.

**Table 1:** Performance scores (average over classes) for model predictions

Model	AUC	CA	F1	Precision	Recall
*10 K features selected by* ANOVA					
Gradient boosting	0.983	0.878	0.872	0.876	0.878
Random forest	0.934	0.728	0.707	0.703	0.728
AdaBoost	0.764	0.598	0.596	0.599	0.598
CN2 rule inducer	0.817	0.585	0.583	0.586	0.585
k Nearest neighbor	0.785	0.479	0.422	0.502	0.479
Neural network	0.643	0.432	0.415	0.415	0.432
Support vector machine	0.779	0.445	0.395	0.476	0.445
Naive bayes		0.143	0.164	0.269	0.143
Logistic regression	0.157	0.067	0.081	0.136	0.067
Stochastic gradient descent	0.425	0.025	0.028	0.031	0.025
*10 K features selected by gain ratio*					
Gradient boosting	0.986	0.903	0.895	0.895	0.903
Random forest	0.959	0.811	0.786	0.779	0.811
AdaBoost	0.838	0.725	0.721	0.721	0.725
CN2 rule inducer	0.860	0.672	0.672	0.673	0.672
Neural network	0.723	0.518	0.506	0.509	0.518
k nearest neighbor	0.802	0.510	0.461	0.639	0.510
Support vector machine	0.847	0.496	0.429	0.494	0.496
Naive bayes		0.361	0.389	0.464	0.361
Logistic regression	0.114	0.078	0.085	0.153	0.078
Stochastic gradient descent	0.422	0.032	0.033	0.036	0.032

*AUC* area under curve, *CA* classification accuracy

We next examined model performance across individual cancer types. Figure 2B shows the confusion matrix for the organ-specific classification results obtained from Gradient Boosting with the actual cancer types. For instance, in the case of prediction based on features selected by ANOVA, of the 132 cases predicted to be breast cancer (BRCA) samples, 97.5% of the cases predicted were actual BRCA cases (Fig. 2B). Similarly, for the cases classified according to the features selected by Gain Ratio, 121 cases were predicted as BRCA cases and 96.7% were actual BRCA cases. Overall, predictability was good with both methods for cancers with higher numbers of cases available for training (> 70) but was low for cancers with fewer than 20 samples in the training set (GBM, HNSC, and STAD). These results show that reducing the number of CpG sites using filter-based methods of feature extraction yields favorable classification results when using a training set with > 70 cases.

### The prediction accuracy of features selected using an embedded machine-learning classifier

**Fig. 3:**
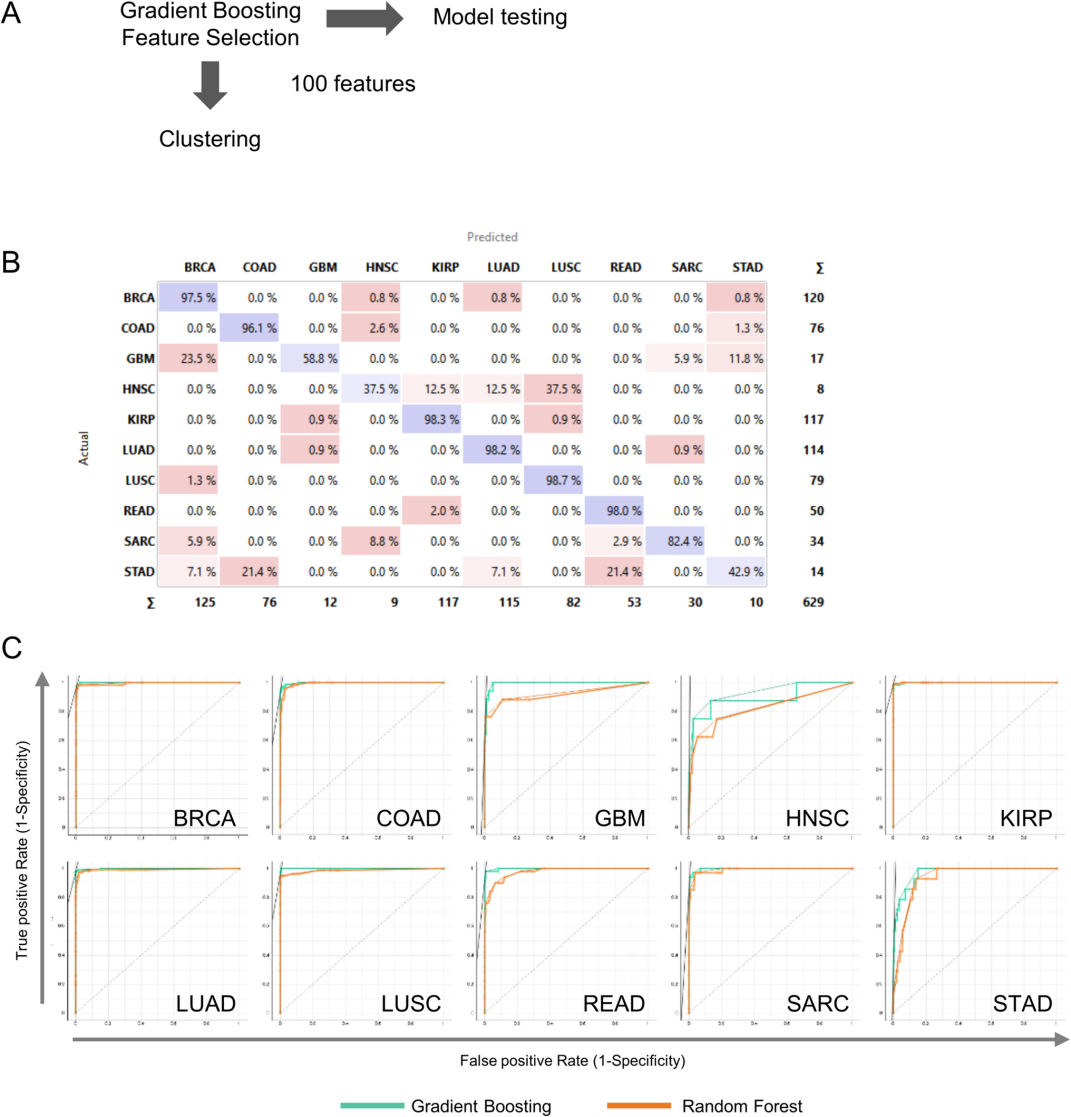
Evaluation of the prediction model using features selected by a gradient-boosting feature ranker. **A** Flowchart of the analysis process. **B** Confusion matrix showing the percentage of patients actually predicted by Gain Ratio classifier trained features selected by Gradient Boosting as a feature ranker. **C** Receiver operating characteristic (ROC) curve analysis of cancer type prediction from Gain Ratio and Random Forest model for each cancer type

Despite the accuracy of feature selection with ANOVA and Gain Ratio, these methods still required many features to train the classifiers. This is a critical problem and making these feature sets unfeasible for creating a targeted focused panel. Machine learning algorithms can improve feature selection by removing irrelevant or redundant features to reduce the dimensionality of inputs, thus improving the performance of training and learning models. To test this approach, we used Gradient Boosting as a base learner to rank features for prediction modeling and unsupervised clustering analysis (Fig. 3A). One hundred features were extracted in the feature selection process. The model was subjected to stratified fivefold cross-validation and performance evaluation as before. Overall performance scores from the top three performing algorithms were comparable to those of ANOVA and Gain Ratio (Table 2). Performance across individual cancers was similar between this model and those of ANOVA and Gain Ratio for cancers with > 70 cases and was improved for cancers with few samples (< 20) in the training set (Fig. 3B). We next compared Gradient Boosting classification with Random Forest, as it is also an ensemble decision tree-based model but differs in how it builds its trees. Random Forest was also the best-performing model after Gradient Boosting. A comparison of the two models is shown as receiver operating characteristic (ROC) curves for each tumor type in Fig. 3C. These findings show that the classification of tumors based on 100 features selected with Gradient Boosting performed similarly to filter models that required 10,000 features (Supplementary Table S3). Our selection approach led to the development of a computationally inexpensive classification model.

**Table 2:** Performance scores (average over classes) for model predictions

Model	AUC	CA	F1	Precision	Recall
*100 features selected by gradient boosting*					
Gradient boosting	0.983	0.878	0.872	0.876	0.878
Random forest	0.934	0.728	0.707	0.703	0.728
AdaBoost	0.764	0.598	0.596	0.599	0.598
k nearest neighbor	0.817	0.585	0.583	0.586	0.585
Neural network	0.785	0.479	0.422	0.502	0.479
Support vector machine	0.643	0.432	0.415	0.415	0.432
Naive bayes	0.779	0.445	0.395	0.476	0.445
Stochastic gradient descent		0.143	0.164	0.269	0.143
Logistic regression	0.157	0.067	0.081	0.136	0.067

*AUC* area under curve, *CA* classification accuracy

### Unsupervised analysis of features selected by Gradient Boosting

**Fig. 4:**
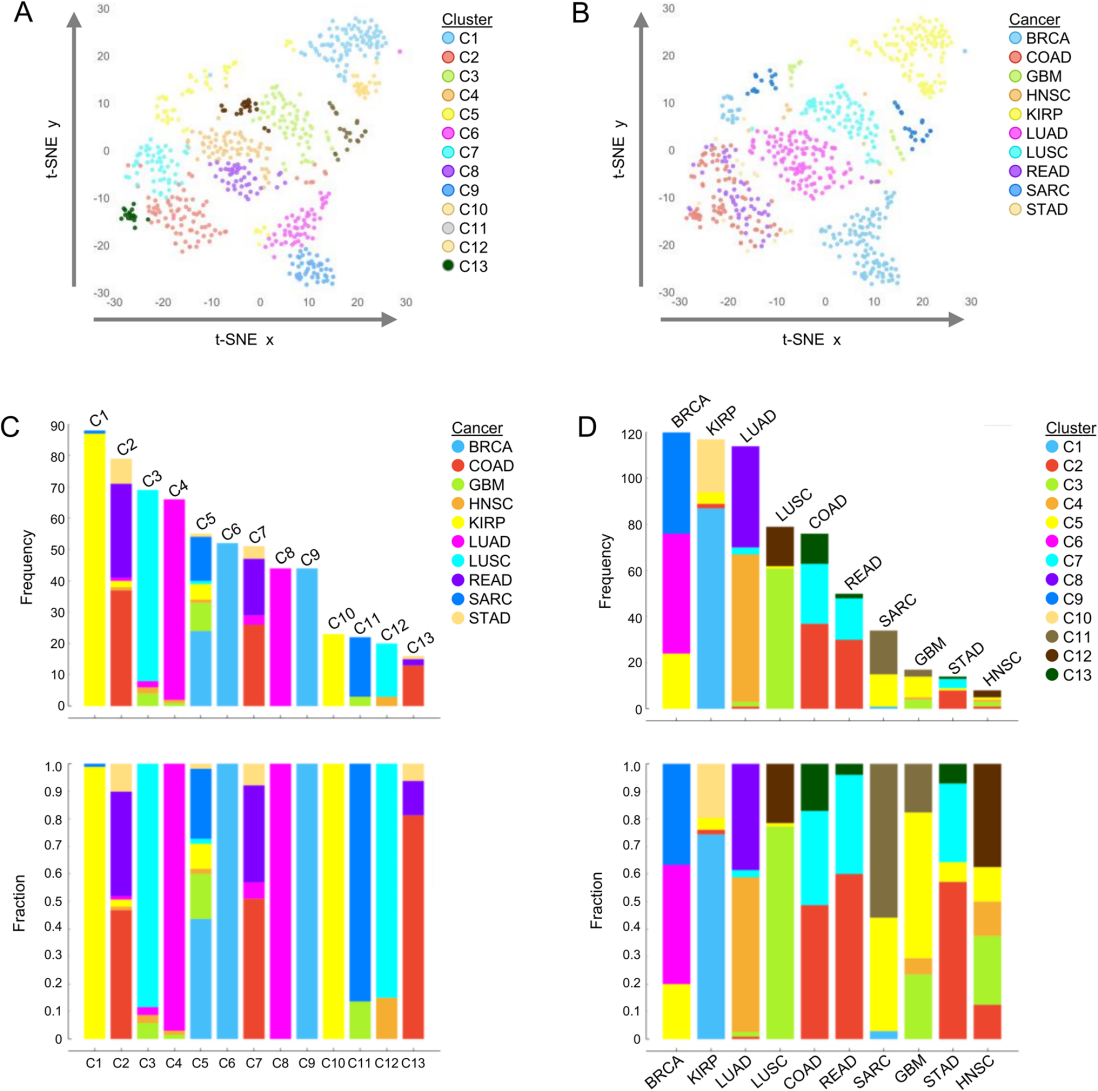
Characterization of the 100 features selected by Gradient Boosting in the training model. Visualization of data using t-distributed stochastic neighbor embedding (t-SNE) of patients (n = 629) based on the methylation of CpG sites selected by Gradient Boosting. The Louvain method of community detection was used to identify patient clusters. Colors were assigned according to cluster (**A**) or cancer type (**B**). Bar plots showing the frequency and relative fraction of patient associations between cluster (**C**) and cancer type (**D**)

For the unsupervised analyses, we first performed community detection and clustering of patients based on the 100 CpG sites selected by the Gradient Boosting learner. Thirteen clusters were identified and 2D visualization of these clusters by t-SNE shows distinct clustering that is much more closely correlated with cancer types compared to those observed by clustering from ANOVA or Gain Ratio feature selection methods (Fig. 4A, B). Moreover, it was visually obvious some cancers were associated with more than one cluster. For example, BRCA was closely associated with clusters C6 and C9, whereas LUAD was primarily associated with clusters C4 and C8, and KIRP was mostly associated with clusters C1 and C10. These results suggest that this approach may detect cancer subtypes. We further explored clusters and their relationship to primary cancers. Cluster C1 included the largest number of patients (n = 88) which comprised 14% of the total population, and cluster C13, the smallest, contained 16 cases representing 2.5% of the population (Fig. 4C). Seven clusters contained at least 50 cases, clusters C1-C7. The associations between the cancer site and clusters varied. Clusters C8, C9, and C10 were unique to LUAD, BRCA, and KIRP, respectively. Conversely, clusters C2, C3, C5, and C7 were more heterogenous and were associated with 4 or more cancers. We also examined the association between cancer type and cluster (Fig. 4D). For the most part, all cancers were associated with three or more clusters. Cases from LUAD and HNSC showed the greatest heterogeneity and were linked to five clusters. On the other hand, KIRP and LUSC showed less heterogeneity with over 75% of the cases from a single cluster (Fig. 4D). Interestingly, we also found COAD, READ, and STAD to be similar to each other, being comprised primarily of clusters C2 and C7. Ninety-five percent (75/79) of the cases in C2 and 94% (48/51) of cases in C7 were associated with GI cancers (COAD, READ, and STAD). Another important observation was in BRCA, where 43.3% (52/120) and 36.7% (44/120) of the cases were associated with clusters C6 and C9, respectively. Both clusters were unique to BRCA. The remaining 20% (24/120) of cases were linked to cluster C5, a heterogeneous cluster that was associated with seven cancer types. These findings indicate that the selected features may allow the differentiation of cancer subtypes and even group molecularly similar cancers.

**Fig. 5:**
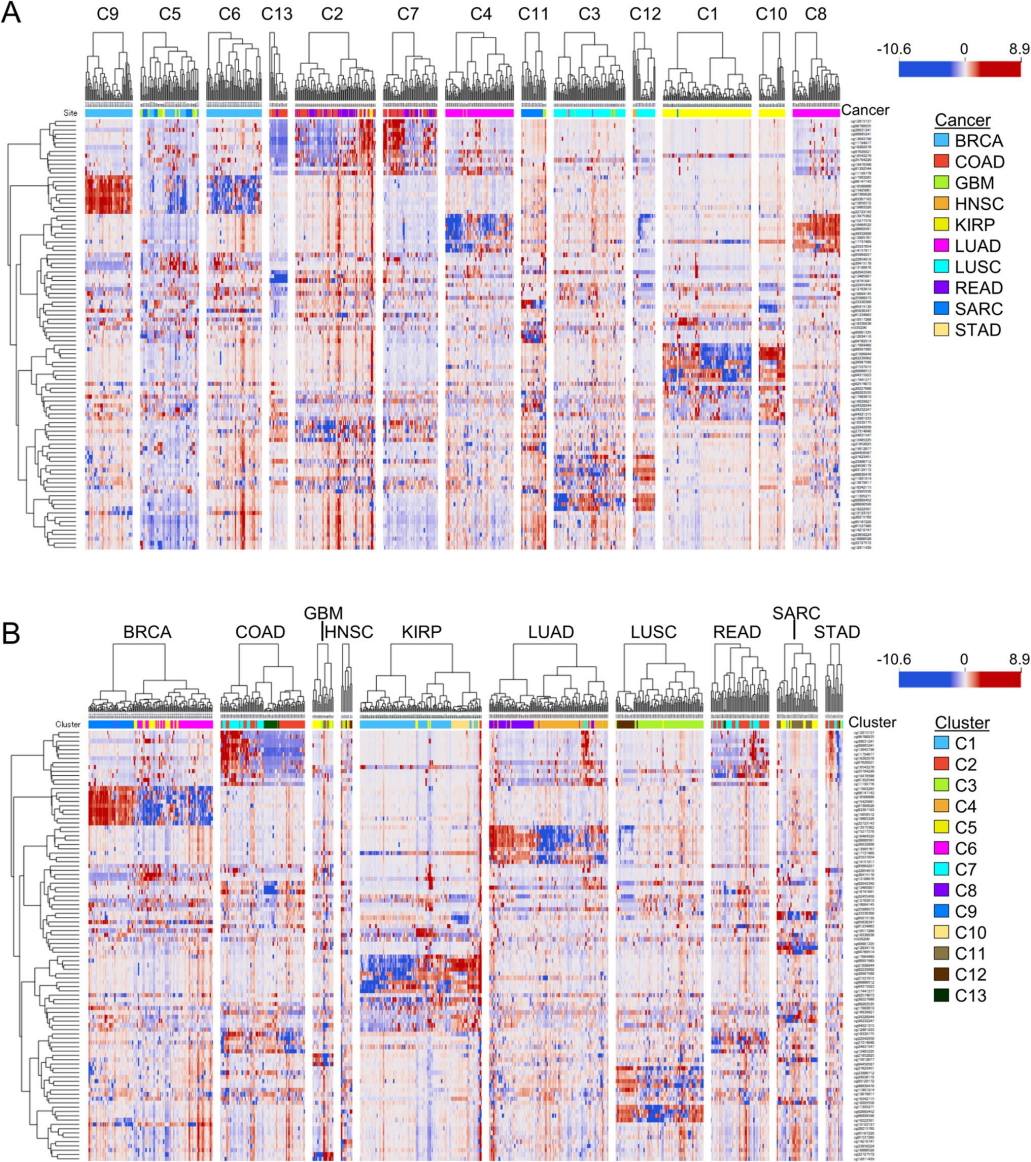
Unsupervised hierarchical clustering analysis of methylation profiling for CpG sites selected by Gradient Boosting. Hierarchically clustered heatmaps of patients (n = 629) from the training set and 100 CpG sites selected by Gradient Boosting split according to Louvain cluster (**A**) or cancer type (**B**). Dendrograms represent Euclidean distances for CpG sites and Pearson correlation coefficients for patients. Hierarchical clustering is based on the Ward linkage method. The scale bar represents relative levels of methylation, the red color indicates high levels, while the blue color represents low levels

Therefore, we next examined the relationships between the features selected by Gradient Boosting. For this, we performed an unsupervised correlation analysis of the 100 CpG sites and found high correlations between some CpG sites (Supplementary Fig. S2). We next analyzed the methylation status of these CpG sites with both cluster and cancer types using hierarchically clustered heatmaps. From these heat maps, we can see quite distinct patterns within the clusters (Fig. 5A). For instance, the CpG sites in cluster C5 tended to have mostly low β values., whereas these were largely high β values in cluster C11. Other clusters showed a distinct pattern of higher/lower β value of methylation. Clusters C9 and C6 were among the clusters that displayed distinct higher/lower β value in certain CpG sites. We next examined methylation status in cases clustered and grouped according to cancer type (Fig. 5B). Compared to Fig. 5A, this examination revealed a different pattern in the clustering of methylation in CpG sites. In several cancers, such as BRCA, KIRP, COAD, and LUAD, distinct subgroups were identified based on variations in higher β values within specific CpG sites. Additionally, gastrointestinal (GI) cancers (COAD, READ, and STAD) exhibited similar methylation patterns. Our findings suggest that the methylation profiles of CpG sites from our feature set could be linked to certain biological characteristics that define molecular cancer subtypes.

### Validation of the prediction model

**Fig. 6:**
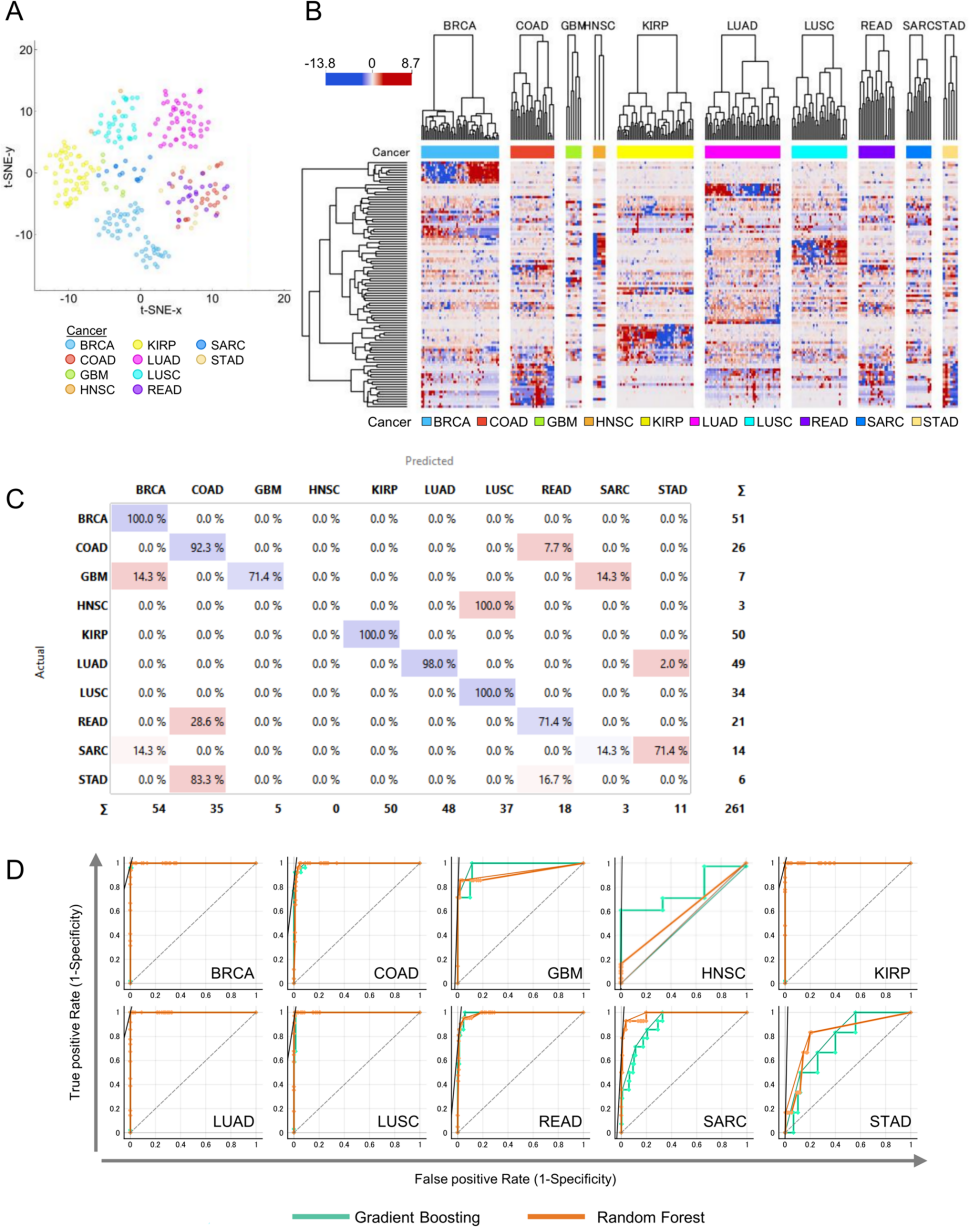
Validation of cancer-type prediction performance using a focused set of CpG sites selected by Gradient Boosting and examination of features. The validity of the prediction model was evaluated using a test set of 261 cases from the TCGA dataset. **A** Visualization of data using t-distributed stochastic neighbor embedding (t-SNE) of patients based on the methylation of 100 CpG sites selected by Gradient Boosting. **B** Hierarchically clustered heatmaps of patients in the test set and 100 CpG sites selected by Gradient Boosting split according to cancer. Dendrograms represent Euclidean distances for CpG sites and Pearson correlation coefficients for patients. Hierarchical clustering is based on the Ward linkage method. The scale bar represents relative levels of methylation, the red color indicates high levels, while the blue color represents low levels. **C** Confusion matrix showing the percentage of patients actually predicted by Gain Ratio classifier trained features selected by Gradient Boosting as a feature ranker. **D** Receiver operating characteristic (ROC) curve analysis of cancer type prediction from Gain Ratio and Random Forest model for each cancer type

Our final goal was to evaluate the predictability and performance of the models built on the training set. For this, we used a test set (n = 261), comprised of pre-partitioned data from the TCGA dataset. Initial 2D visual analysis of samples using t-SNE after data preprocessing showed a distinct grouping of cases that were largely associated with cancer type (Fig. 6A).

Further unsupervised evaluation of the methylation levels for the selected methylation sites revealed expression profiles that resembled those of the test set (Fig. 6B). These patterns were particularly evident with BRCA, KIRP, and LUAD. Similar to the training set, the GI cancers (COAD, READ, and STAD) of the validation set had similar methylation patterns that resembled each other.

We next examined the predictability of the test using Gradient Boosting as a trained model and compared it with other machine learning models. A summary of the test results is shown in Table 3.

**Table 3:** Performance scores (average over classes) for model predictions

Model	AUC	CA	F1	Precision	Recall	Model category
Gradient boosting	0.974	0.877	0.867	0.882	0.877	Ensemble-boosting
XGBoost (XGB)	0.981	0.912	0.897	0.929	0.912	Ensemble-boosting
CatBoost	0.994	0.935	0.917	0.908	0.935	Ensemble-boosting
AdaBoost	0.887	0.816	0.784	0.791	0.816	Ensemble-boosting
Random forest (RF)	0.985	0.897	0.878	0.878	0.897	Ensemble-bagging
XGB-RF	0.946	0.828	0.814	0.834	0.828	Ensemble-hybrid
Neural network	0.964	0.801	0.784	0.791	0.801	Neural Nets
CN2 rule inducer	0.946	0.843	0.834	0.829	0.843	Rule System
Stochastic gradient descent	0.570	0.276	0.258	0.244	0.276	Iterative
Ridge regression	0.499	0.211	0.159	0.131	0.211	Regression
LASSO	0.488	0.218	0.154	0.137	0.218	Regression
Logistic regression	0.500	0.207	0.162	0.182	0.207	Classification
k nearest neighbor	0.931	0.774	0.737	0.783	0.774	Classification
Naive bayes	0.995	0.766	0.793	0.890	0.766	Classification
Support vector machine	0.961	0.743	0.693	0.737	0.743	Classification

*AUC* area under curve, *CA* classification accuracy, *XGBoost* eXtreme Gradient Boosting, *LASSO* least absolute shrinkage and selection operator

Overall, the performance of Gradient Boosting remained relatively good with an average classification accuracy of 0.877 and an F1 score of 0.867. Of all the models examined, CatBoost, which is an ensemble-boosting model, had the best performance with an F1 score of 0.917. Random forest is another popular ensemble model that uses bagging (i.e., bootstrap aggregation) as a concept to generate trees and also performed well with an F1 score of 0.878. We next examined the performance of Gradient Boosting across individual cancer predictions. All cases for BRCA, KIRP, and LUSC were predicted correctly, with three false positives for BRCA and LUSC (Fig. 6C). Cancers such as COAD and LUAD were correctly predicted > 90%. Seventy-one percent of READ cases were correctly predicted and 28.6% of the cases were predicted as COAD, which has some anatomical and transcriptomic similarities to READ [26]. Overall cancers with low training samples also performed poorly with predictions. Only 14.3% of SARC cases were correctly classified. Six patients were incorrectly classified but were predicted to be either COAD or READ. All three HNSC patients were predicted to be LUSC cases. Lastly, we wanted to compare the performance of Gradient-Boosting predictions with that of Random Forest. For this, we generated ROC plots for each cancer type (Fig. 6D). The performance of Gradient Boosting and Random Forest in the area under the curve (AUC) was comparable for the selected cancer types. Overall, our results are promising for the predictive potential of our feature selection model and provide the basis for developing and constructing targeted methylation profiling to identify the origins of CUP.

## Discussion

Our primary goal was to establish a system that would aid in predicting the origin of CUP from a focused methylation profiling panel. The initial step was to develop a method for extracting the most relevant features and then constructing and testing a prediction model based on that set of features. We chose to test our methodology using representative cancer cases because of the large number of features available from the Illumina Infinium Human Methylation 450 k platform. As a proof-of-concept study, we also wanted to limit the number of samples to maximize computing resources. We also wanted to ensure that we could create a prediction model that could be trained on and distinguish challenging primary cancer types *i.e.*, rare, heterogeneous. Here, we demonstrate the use of a machine learning approach to construct a targeted DNA methylation-based profiling model that can classify and predict cancer types. Our approach enabled us to extract relevant methylation data based on β scores for the entire genome from selected cancers in the TCGA dataset. Our machine-leaning model could classify tumors based on a methylation profile that consisted of 100 methylation sites. This classifier set represents a mere 0.02% of the total available from the original methylation profiling array and was extracted using a machine learning learner used as a feature ranker.

DNA methylation is an important epigenetic process by which gene expression is repressed by the transfer of a methyl group onto the C5 position of the cytosine to form 5-methylcytosine [27]. Epigenetic programs define a normal cell’s identity and function, whereas alterations to DNA methylation, histone modification, microRNAs, and nucleosomes contribute to carcinogenesis [28]. CpG methylation plays an important role in the regulation of gene expression and is intimately involved in cancer development and progression and aberrant DNA methylation is one of the hallmarks of cancers. Genome-wide analyses of DNA methylation profiles in human tissue have revealed complex but tissue-specific [29–32] patterns in DNA methylation that carry over into cancers. Each organ has a unique methylation pattern, which has been shown to be reflected in cancer cells [33]. However, cancer cells exhibit aberrant DNA methylation patterns compared to their normal tissue counterparts [34]. Additionally, the distinct methylation profile of a tumor is shaped by the complex interplay of various cell types within the tumor microenvironment (TME), including malignant (cancer) cells, stromal cells, and immune cells [35, 36]. Stromal cells play an important role in cancer progression, and the methylation profile of these cells may also reflect cancer characteristics. For example, it has been reported that the methylation pattern of stromal cells in cancer can influence the methylation pattern of cancer cells [37]. DNA methylation profiles also exhibit organ-specific characteristics in cancer cells. For example, in colon cancer, methylation of CpG islands is frequently observed in specific genes, which can be used for cancer diagnosis and prognosis prediction [38]. DNA methylation-specific patterns have also been used to differentiate between cancer subtypes, stages, and grades [39, 40]. Thus, the methylome provides a rich source of data from which cancer biomarkers may be mined. This study aimed to compare CpG methylation profiles across cancer samples from different organs. The objective was to determine if organ-specific CpG methylation patterns are retained among cancer samples and if these profiles could be used to predict cancer type.

Genome-wide DNA methylation profiling studies have identified methylation patterns that could be used as biomarkers for disease subtypes, prognosis, and drug response [41]. The methylome also provides a source of data that could be mined to build cancer-type-specific classification and prediction models—however, this is a very large pool, and the question remains as to which methylation sites would be the most useful. Several researchers have carried out research to develop prediction models that determine cancer type based on methylation profiles [42–47]. The methods used by investigators to extract relevant data and the learning models used to derive predictions have differed between studies. However, most studies still used a high number of features for their training models. For instance, Jurmeister et al*.* used 10,000 CpG sites with the highest standard deviation and a Random Forest classifier to differentiate between pulmonary enteric adenocarcinoma and metastatic colorectal cancer [48]. Another study aimed to identify cancer origins by methylation profiling using 10,360 CpG sites, selected by a combination of statistical methods, as an input layer of neural network classification [45]. A large study by the Circulating Cell-free Genome Atlas (CCGA) Consortium and STRIVE investigators, supported by GRAIL Inc., performed targeted DNA methylation analyses of cell-free DNA (cfDNA) from over 50 cancer types with greater than 90% accuracy. However, their targeted methylation panel covered about 100,000 distinct sites and contained just over 1.16 million CpG sites.

Panels that require a large number of features for prediction pose real-world challenges that are related to the cost, handling, storage, processing, and security of data. Furthermore, large data sets often contain irrelevant or redundant data that add noise, which reduces model accuracy, performance, and computing efficiency. Feature selection is probably the most significant variable in machine learning, and several tools are available. The primary objective of feature selection is to reduce the number of input variables on training data to improve model performance and reduce the computational costs of modeling. Filter methods rank features according to their scores in various statistical tests for their correlation with the class[47]. Filtering methods are commonly used with high-dimensional datasets because they are typically less computationally demanding and are not susceptible to overfitting. However, filter methods are linear and treat features independently, and do not account for interactions of data. Thus, to achieve accurate results, the size of the output training features must be large to compensate for redundant data. This phenomenon was exemplified in our analysis using ANOVA and Gain Ratio.

Traditional feature ranking methods have been used to study large datasets in biology and require less computational power than more contemporary machine learning methods [22, 49–52]. However, datasets that have complex feature interactions and high levels of redundancy still pose a challenge for filter feature selection methods [47]. Modern machine learning algorithms work better with complex high-dimensional data and have grown in popularity in recent years [53]. Here, we used ANOVA and Gain Ratio as representative feature ranking methods to compare with Gradient Boosting. Gradient Boosting is an ensemble of base (weak) learners and is a standard implementation of tree-based models such as classification and regression trees (CART). The weak learners are then combined (boosted) to compose a strong learning model. These ensembles of trees are more predictive in large datasets, and their feature importance scores reflect more complex interactions, which can then be used to extract the most relevant features [54].

Our goal is to establish a focused methylation panel for predicting the CUP tissue of origin, requiring a limited number of predictive CpG sites. In our study, we examined the feasibility and performance of selecting a compact set of features using Gradient Boosting as a feature ranker and compared it with two filter methods. Our results show that this approach allows us to reduce the number of features by 100-fold while still maintaining comparable performance. We tested this specific set of features in a validation set using various machine learning algorithms, and the tree-based ensemble methods performed the best. Our results also showed that features extracted from the larger cancer sets yielded better prediction results (BRCA, COAD, KIRP, LUAD, and LUSC) compared to those from smaller sets (GBM, HNSC, READ, SARC and STAD). Our research illustrates how dataset size impacts model performance and the dangers of employing smaller data sets, particularly those with high heterogeneity.

Further examination of these features revealed interesting methylation patterns that could be associated with certain cancer characteristics that may be clinically relevant. For instance, we observed cancer subtypes with breast, colon, and lung adenocarcinomas, lung squamous cell carcinomas, and kidney renal papillary cell carcinomas. Conversely, we observed similarities between the methylation profiles among subsets of gastrointestinal cancers, including colon, rectal, and stomach adenocarcinomas. Our 100-feature panel has not only demonstrated accuracy in feature selection but has also revealed that methylation patterns differ among cancer types, similarities exist between cancer types, and subgroups exist within cancer types. These observations suggest that methylation profiles based on our feature set may be related to certain aspects that define established molecular cancer subtypes. which could provide useful information to aid in the treatment stratification of patients. Our 100 CpG sites feature set provided accuracy for the prediction of cancer type but may be insufficient to determine the biological relevance. The purpose of our model was to select features that would be useful for accurate classification. This reduced many features that were redundant and not informative for classification and prediction. However, this does not mean that these features are not biologically relevant and many of those features were likely associated with co-expression networks. We will examine the co-expression networks of these methylation regions in our future studies and hopefully elucidate their biological and clinical significance.

In the current study, we investigated the construction of an algorithm to identify the primary site based on methylation profiling. However, it is important to consider that assigning site-specific therapy based on primary site prediction may not suffice to improve outcomes because it relies on an unproven assumption. In a previous clinical trial of primary site prediction based on 92-gene cancer classification, subgroup analyses showed that patients with responsive tumor types had improved survival with site-specific therapy [10]. Our group also showed that site-specific therapy based on gene expression profiles is beneficial for patients with responsive tumor types although site-specific therapy based on prediction did not significantly improve 1-year survival compared to empiric therapy [11]. This may be due to differences in the clinical efficacy of site-specific treatment to the predicted primary site. In addition to improving the accuracy of primary site prediction algorithms, it could be necessary to prospectively evaluate efficacy through appropriate clinical trial designs. As with any cancer, identifying a potentially actionable alteration would be beneficial for directing alteration-targeted therapy regardless of tissue origin. Panel sequencing studies have shown that at least one genetic alteration occurs in 65–80% of CUP cases [55, 56]. However, less than a third of patients diagnosed with CUP have potentially targetable genetic alterations [57]. To receive the benefits of site-specific therapy, the remaining patients need to rely on an estimator to determine the primary site. In practical clinical settings, a series of supplementary tests, encompassing genetic background analysis and tissue origin prediction through methylation profiling, could aid in identifying the most effective treatment strategy for each patient with CUP.

There are some limitations to our model regarding its value in predicting tissue of origin. Namely, we limited the number of cancer types to ten and there was a large class imbalance in which cancers. However, this was done by design. We wanted to test our approach using a relatively small set given the large amount of data involved. In addition, this study showed that cancer sets with few samples for training did not perform as well as those with larger numbers, which is valuable information that can be used to better design training sets in the future. In addition, we did not use an independent validation set. Nevertheless, our study established the methodology needed to establish proof-of-concept for our approach for feature extraction and will serve as the foundation to build a model that will include additional cancer types as well as independent validation data sets and prospective validation cohorts.

In conclusion, our study has outlined an approach whereby we used an embedded machine learning algorithm to identify a select set of informative features from complex high-dimension data to train and predict cancer type. By extracting a compact set of relevant CpG sites, a custom panel of methylation sites could be constructed, which could be more feasible for clinical applications. Our follow-up studies will expand our model to include additional cancers and prospectively validate custom panel methylation sites to evaluate clinical performance.

## Supplementary Information

Below is the link to the electronic supplementary material.

Supplementary file1 (EPS 6528 KB) Supplementary Figure S1. Batch normalization of training data. Visualization of data using t-distributed stochastic neighbor embedding (t-SNE) of patients (n=629) based on the methylation of CpG sites before and after batch normalization. BRCA: Breast invasive carcinoma, COAD: Colon adenocarcinoma, GBM: Glioblastoma, HNSC: Head and neck squamous cell carcinoma, KIRP: Kidney renal papillary cell carcinoma, LUAD: Lung adenocarcinoma, LUSC: Lung squamous cell carcinoma, READ: Rectum adenocarcinoma, SARC: Soft tissue sarcoma, STAD: Stomach adenocarcinoma

Supplementary file2 (EPS 6930 KB) Supplementary Figure S2: Correlation matrix of CpG sites selected by Gradient Boosting. Hierarchically clustered correlation matrix heat map showing the Pearson distance between the 100 CpG sites selected by Gradient Boosting
